# The impact of small-group virtual patient simulator training on perceptions of individual learning process and curricular integration: a multicentre cohort study of nursing and medical students

**DOI:** 10.1186/s12909-022-03426-3

**Published:** 2022-05-16

**Authors:** André Mestre, Marek Muster, Ahmed Rhassane El Adib, Hugrun Ösp Egilsdottir, Kirsten Røland Byermoen, Miguel Padilha, Thania Aguilar, Nino Tabagari, Lorraine Betts, Leila Sales, Pedro Garcia, Luo Ling, Hugo Café, Alexandra Binnie, Ana Marreiros

**Affiliations:** 1 Faculty of Medicine and Biomedical Sciences, University of Algarve, Campus de Gambelas, Ed. 2 – Norte, 8005-139 Faro, Portugal; 2 Institute of Health Sciences, Medical College of University of Rzeszow, University of Rzeszow, Rzeszow, Poland; 3 Faculty of Medicine and Pharmacy, Cadi Ayyad University, Marrakesh, Morocco; 4 Science Centre Health and Technology, Faculty of Health and Social Sciences, University of South-Eastern Norway, Drammen, Norway; 5 Porto Nursing School, CINTESIS-Tech4edusim, Center for Health Technology and Services Research, University of Porto, Porto, Portugal; 6 Central American Technological University (UNITEC), Tegucigalpa, Honduras; 7 AIETI Medical School, David Tvildiani Medical University, Tbilisi, Georgia; 8 Sally Horsfall Eaton School of Nursing, Waterfront Campus, George Brown College, Toronto, ON Canada; 9 Red Cross Higher School of Health, Lisbon, Portugal; 10 Faculty of Medical Sciences, Nova Medical School, Lisbon, Portugal; 11 Guangxi Medical College, Nanning, Guangxi China; 12 ABC-RI, Algarve Biomedical Center Research Institute, Faro, Portugal; 13 William Osler Health System, Brampton, ON Canada

**Keywords:** Virtual Patient Simulator, Curricular integration, Simulation training, Clinical education, Learning feedback

## Abstract

**Background:**

The COVID-19 pandemic has precipitated rapid changes in medical education to protect students and patients from the risk of infection. Virtual Patient Simulators (VPS) provide a simulated clinical environment in which students can interview and examine a patient, order tests and exams, prioritize interventions, and observe response to therapy, all with minimal risk to themselves and their patients. Like high-fidelity simulators (HFS), VPS are a tool to improve curricular integration. Unlike HFS, VPS require limited infrastructure investment and can be used in low-resource settings. Few studies have examined the impact of VPS training on clinical education. This international, multicenter cohort study was designed to assess the impact of small-group VPS training on individual learning process and curricular integration from the perspective of nursing and medical students.

**Methods:**

We conducted a multi-centre, international cohort study of nursing and medical students. Baseline perceptions of individual learning process and curricular integration were assessed using a 27-item pre-session questionnaire. Students subsequently participated in small-group VPS training sessions lead by a clinical tutor and then completed a 32-item post-session questionnaire, including 25 paired items. Pre- and post-session responses were compared to determine the impact of the small-group VPS experience.

**Results:**

Participants included 617 nursing and medical students from 11 institutions in 8 countries. At baseline, nursing students reported greater curricular integration and more clinical and simulation experience than did medical students. After exposure to small-group VPS training, participants reported significant improvements in 5/6 items relating to individual learning process and 7/7 items relating to curricular integration. The impact of the VPS experience was similar amongst nursing and medical students.

**Conclusions:**

In this multi-centre study, perceptions of individual learning process and curricular integration improved after exposure to small-group VPS training. Nursing and medical students showed similar impact. Small-group VPS training is an accessible, low-risk educational strategy that can improve student perceptions of individual learning process and curricular integration.

**Supplementary Information:**

The online version contains supplementary material available at 10.1186/s12909-022-03426-3.

## Background

Virtual Patient Simulators (VPS) (also known as “High Fidelity Software Simulations” [1] or “Clinical Virtual Simulators” [2]) are computer-based programs in which students emulate the role of health care providers while caring for virtual patients. Learners can interview and examine their patient, order exams, prioritize and choose interventions and observe response to therapy in real-time.

**Fig. 1:**
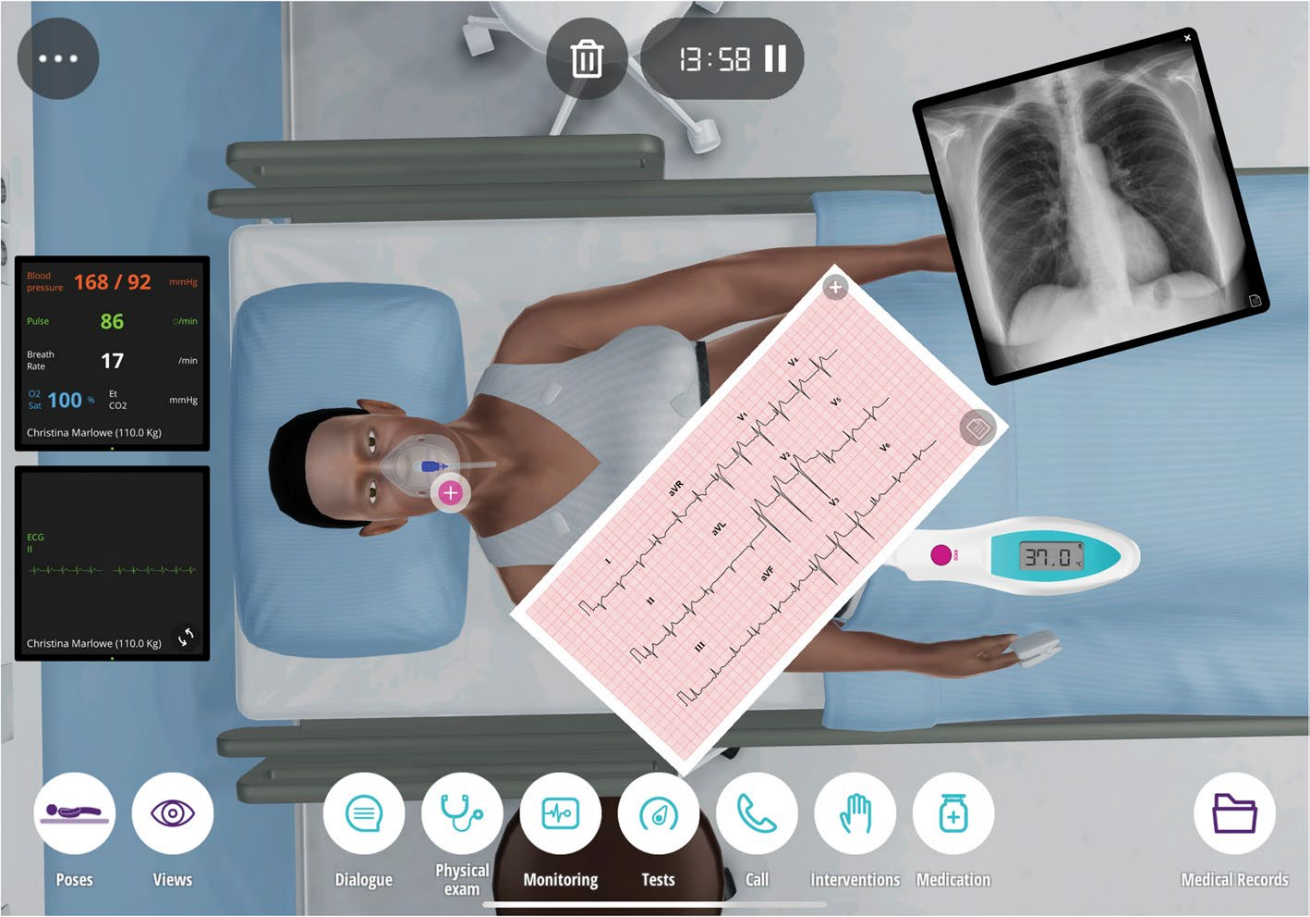
The graphical interface of the Body Interact™ VPS displays the virtual patient and allows the learner to interact with the patient and their clinical environment

VPS evolved from Virtual Patients (VP), which are static, computer-based patient cases that provide clinical information in text and image format and then ask for a diagnosis or treatment plan [1]. Unlike a VP case, however, the VPS emulates an actual clinical encounter with the help of a graphical interface (Fig. 1) that enables the learner to interact with the virtual patient and their clinical environment. Thus, VPS are an interactive training tool that can mimic a “hands-on” clinical encounter. Like high fidelity simulators (HFS), VPS allow students to apply their learned knowledge to clinical problems in a structured, low risk setting [3], thereby integrating basic and clinical learning [4]. However, VPS are less expensive than HFS [5, 6], can be used both individually and in groups and are available in practically any setting in which there is computer access.

Although VPS are intuitively appealing as an educational tool, studies of VPS and their impact on clinical education are lacking [7, 8]. A study of Portuguese nursing students reported that the Body Interact™ VPS was easy to use and relevant [2]. A subsequent study randomized 42 nursing students to a VPS educational session vs a low-fidelity simulation session; students who attended the VPS session showed improved knowledge retention and clinical reasoning [9]. A study of Norwegian nursing students reported that the Laerdal vSim for Nursing™ VPS was also well-received amongst nursing students [10] and a study from China reported that a vSim for Nursing teaching session improved knowledge scores amongst nursing students when compared with the usual clinical curriculum [11]. These studies highlight that VPS are easy to use and can help with knowledge acquisition and retention. However, studies looking at the impact of VPS on individual learning and curricular integration are lacking.

In this study we analysed the impact of small-group VPS training on perceptions of individual learning process and curricular integration amongst nursing and medical students.

## Methods

We conducted an international, multi-center cohort study to analyse the impact of small-group VPS training on perceptions of individual learning process and curricular integration. Thirty nursing and medical schools with access to the Body Interact™ VPS were invited to participate in the study, of which eleven agreed to participate: Faculty of Medicine and Biomedical Sciences, University of Algarve, Faro, Portugal; Institute of Health Sciences, Medical College of University of Rzeszow, University of Rzeszow, Poland; Cadi Ayyad University, Marrakesh, Morocco; University of South-Eastern Norway, Drammen, Norway; Porto School of Nursing School, Porto, Portugal; Central American Technological University, Tegucigalpa Honduras; David Tvildiani Medical University, AIETI Medical School, Tbilisi, Georgia; George Brown College Sally Horsfall Eaton School of Nursing, Toronto, Canada; Red Cross Higher School of Health, Lisbon, Portugal; NOVA Medical School, Lisbon, Portugal and Guangxi Medical College, Guangxi, China. The study took place between November 2019 and July 2020. Ethics approval was obtained at participating institutions, in accordance with local regulations. Written consent was obtained from all participants.

### Protocol implementation

The study protocol was defined by the investigators and implemented at each site by one or more clinical tutors with VPS teaching experience (see Supplementary methods for the implementation protocol). The protocol was designed to be pragmatic, given the varying conditions at different participating institutions. All sites had access to the Body Interact™ VPS in interactive table or computer format and compliance with the protocol was ensured through emails, online meetings, telephone contact and activity registration. Participants were nursing or medical students with no prior contact with the VPS. In keeping with the pragmatic design, the small-group VPS training sessions could be integrated into the regular curriculum or offered as a stand-alone session, at the discretion of the tutor(s). Student participation in the study was voluntary.

Prior to the VPS training session, participants received a brief, non-judgemental introduction to the VPS with an opportunity to ask questions. After this session, the pre-session questionnaire (U0) was completed via an online form. Small-group VPS training sessions took place in groups of 5 to 9 students and consisted of a sample clinical case, to familiarize the students with the functionality of the VPS, followed by two additional clinical cases chosen by the tutor. After the session, participants completed the post-session questionnaire (U1) via an online form. Only participants who completed both questionnaires were included in the final sample. Data access, handling, and treatment were carried out exclusively by the authors.

### Development of the assessment instrument

The study-specific instrument was developed after literature review and consultation with 13 health education experts with medical and nursing backgrounds. The pre-session questionnaire (U0, Supplementary Table 1) and the post-session questionnaire (U1, Supplementary Table 2) assessed three empirical dimensions: (1) individual learning process (2) degree of curricular integration of the student’s course and (3) the student’s perception of (a) the VPS environment and (b) the impact of VPS training on student learning. Twenty-five paired items appeared on the pre- and post-questionnaires while two items were exclusive to the pre-questionnaire (U0.7 and U0.8) and seven items were exclusive to the post-questionnaire (U1.26 to U1.32). All items were assessed on a 7-point Likert scale. Scores of 1–3 were interpreted as “negative”, 4 was considered “neutral” and 5–7 were interpreted as “positive”.

Reliability coefficients for the pre- and post-session questionnaires were determined via iterative integration of medical and nursing schools, resulting in an *alpha Cronbach* (*α)* of 0.945 for U0 and 0.949 for U1, confirming good reliability of the instruments.

### Statistical analysis

Statistical analysis was performed using *IBM SPSS STATISTIC software, version 24*. All items were descriptively analysed. Changes in paired items on the pre- and post-session questionnaires were assessed using the paired-samples *t*-test. Statistical significance was considered *P* < 0.01. Factor analysis was performed for both the pre- and post-session questionnaires using the Principal Component method. Differences between nursing and medical students were assessed using the *t*-test for independent samples. Correlations were determined using the Pearson correlation coefficient.

## Results

Medical and nursing students from 11 educational institutions were recruited to participate in the study. Of the 681 students who participated in the small-group VPS training sessions, 617 (90.6%) consented to participate in the study and completed both study questionnaires. Study participants consisted of 324 (52.5%) nursing students and 293 (47.5%) medical students. The average age of participants was 23 years (*SD* = 3.8) and females outnumbered males by 482 (78.1%) to 135 (21.9%). Amongst participants for whom year of study was known, 500 (91.6%) were in their pre-clinical years (corresponding to years 1–2 for nursing students and years 1–3 for medical students) while 46 (8.4%) were in their clinical years (Supplementary Fig. 1).

### Pre-session questionnaire

On the pre-session questionnaire, participants were asked about their individual learning process (Table 1: questions U0.1 to U0.6). Nursing students reported greater balance between theoretical studies and the practical application of knowledge (*P* < 0.001), more development of communication skills (*P* < 0.001), greater confidence in their knowledge and in the decision-making process (*P* < 0.001) and more development of group and conflict management skills (*P* < 0.001) than did medical students (Supplementary Fig. 2). Conversely, medical students reported that their studies were more focused on theory (*P* < 0.001). Overall, students felt that their clinical experience (*M* = 4.61, *SD* = 1.53) and simulation experience (*M* = 4.65, *SD* = 1.59) were appropriate for their knowledge level, however nursing students rated their clinical and simulation experience more highly than did medical students (*P* < 0.001 and *P* < 0.001, respectively) (Table 1).

**Table 1:** Results of the Pre-Session Questionnaire (U0)

U0 | Pre-Session with Body Interact simulator		Total (*n* = 617)	Medicine (*n* = 293)	Nursing (*n* = 324)	*t*-test
**Items scored from 1 = totally disagree to 7 = totally agree**		**Mean (SD)**			***P***
U0.1	I am able to organize my reasoning	5.13(1.21)	5.09(1.24)	5.16(1.18)	.47
U0.2	My studies are mainly focused on theory	4.69(1.44)	5.01(1.38)	4.39(1.44)	.001
U0.3	My studies balance theoretical studies with the practical application of knowledge	5.05(1.46)	4.57(1.58)	5.49(1.19)	.001
U0.4	My learning process allows for suitable development of my communication skills	4.95(1.36)	4.62(1.51)	5.24(1.14)	.001
U0.5	My learning process allows me to build my confidence (in my knowledge and in the decision-making process)	5.06(1.41)	4.82(1.53)	5.28(1.25)	.001
U0.6	My learning process allows me to develop my skills in group management and conflict management	4.94(1.38)	4.73(1.50)	5.13(1.24)	.001
U0.7	My clinical experience is appropriate for my knowledge level	4.61(1.53)	4.29(4.90)	4.90(1.41)	.001
U0.8	My simulation experience is appropriate for my knowledge level	4.65(1.59)	4.41(4.88)	4.88(1.37)	.001
U0.9	In my course the contents are well integrated and connected with each other	5.03(1.34)	4.79(1.43)	5.26(1.20)	.001
0.10	In my course there are opportunities to apply new learning to practical clinical cases	5.18(1.36)	4.87(1.51)	5.46(1.14)	.001
U0.11	In my course we have the opportunity to participate in clinical simulations	5.25(1.64)	4.70(1.80)	5.75(1.29)	.001
U0.12	In my course there is adequate training in communication techniques	4.80(1.53)	4.34(1.67)	5.22(1.24)	.001
U0.13	In my course there is discussion/debate of clinical decisions in a controlled learning environment	5.06(1.45)	4.72(1.56)	5.37(1.26)	.001
U0.14	My course helps me build the personal confidence necessary to function as a future professional	5.04(1.45)	4.75(1.60)	5.31(1.25)	.001
U0.15	I consider the teaching methods in my course appropriate	5.04(1.42)	4.71(1.58)	5.34(1.17)	.001
**Items scored from 1 = low to 7 = high**					
U0.16	Expectations regarding the use of a new learning tool	5.88(1.21)	6.03(1.17)	5.75(1.23)	.005
U0.17	Expectations regarding the use of a technological resource for learning	5.79(1.22)	6.05(1.14)	5.76(1.27)	.003
U0.18	Expectations regarding using Body Interact as a simulator	5.81(1.24)	6.10(1.11)	5.79(1.25)	.001
U0.19	I expect that Body Interact will help to fill in the learning gaps in the teaching process	5.82(1.21)	6.00(1.13)	5.64(1.25)	.001
U0.20	I expect that Body Interact will help to fill in the individual gaps in my current learning	5.79(1.22)	6.01(1.09)	5.58(1.29)	.001
U0.21	I expect that Body Interact will provide real feedback on my learning	5.81(1.24)	6.07(1.06)	5.59(1.35)	.001
U0.22	I expect that Body Interact will help me identify individual weaknesses in my competencies	5.89(1.16)	6.04(1.09)	5.75(1.20)	.002
U0.23	I expect that Body Interact will give me clinical experience (through simulation)	5.82(1.21)	6.00(1.16)	5.66(1.23)	.001
U0.24	I expect that Body Interact will validate the competencies I have already acquired (through simulation)	5.81(1.18)	5.97(1.12)	5.67(1.22)	.002
U0.25	I expect that Body Interact will help me practice decision-making strategies	5.90(1.09)	6.04(1.05)	5.77(1.11)	.003
U0.26	I expect that Body Interact transform clinical decision-making errors into a constructive learning process	5.97(1.13)	6.12(1.09)	5.84(1.15)	.002
U0.27	I expect that that Body Interact will become an important learning tool	5.96(1.20)	6.20(1.11)	5.75(1.24)	.001

Participants were asked about the degree of curricular integration in their courses (Table 1: questions U0.9 to U0.15). Nursing students reported greater integration of course contents (*P* < 0.001), more opportunities to apply learning to practical clinical cases (*P* < 0.001), more opportunities to participate in clinical simulations (*P* < 0.001), more training in communication techniques (*P* < 0.001), and more appropriate teaching methods (*P* < 0.001) than did medical students (Supplementary Fig. 3). Nursing students were also more confident that their courses were helping them build confidence as future professionals (*P* < 0.001) (Table 1).

### Pre-session expectations of the VPS experience

On the pre-session questionnaire, participants were asked about their expectations of the VPS training experience (questions U0.16 to U0.25). Students had high expectations for the VPS as a learning tool (*M* = 5.96, *SD* = 1.20), as a tool to practice decision-making strategies (*M* = 5.90, *SD* = 1.09), and as a tool to transform decision-making errors into a constructive learning process (*M* = 5.97, *SD* = 1.13) (Table 1). Medical students reported higher expectations than did nursing students across all items (Table 1).

### Impact of small-group VPS training on individual learning process

**Fig. 2:**
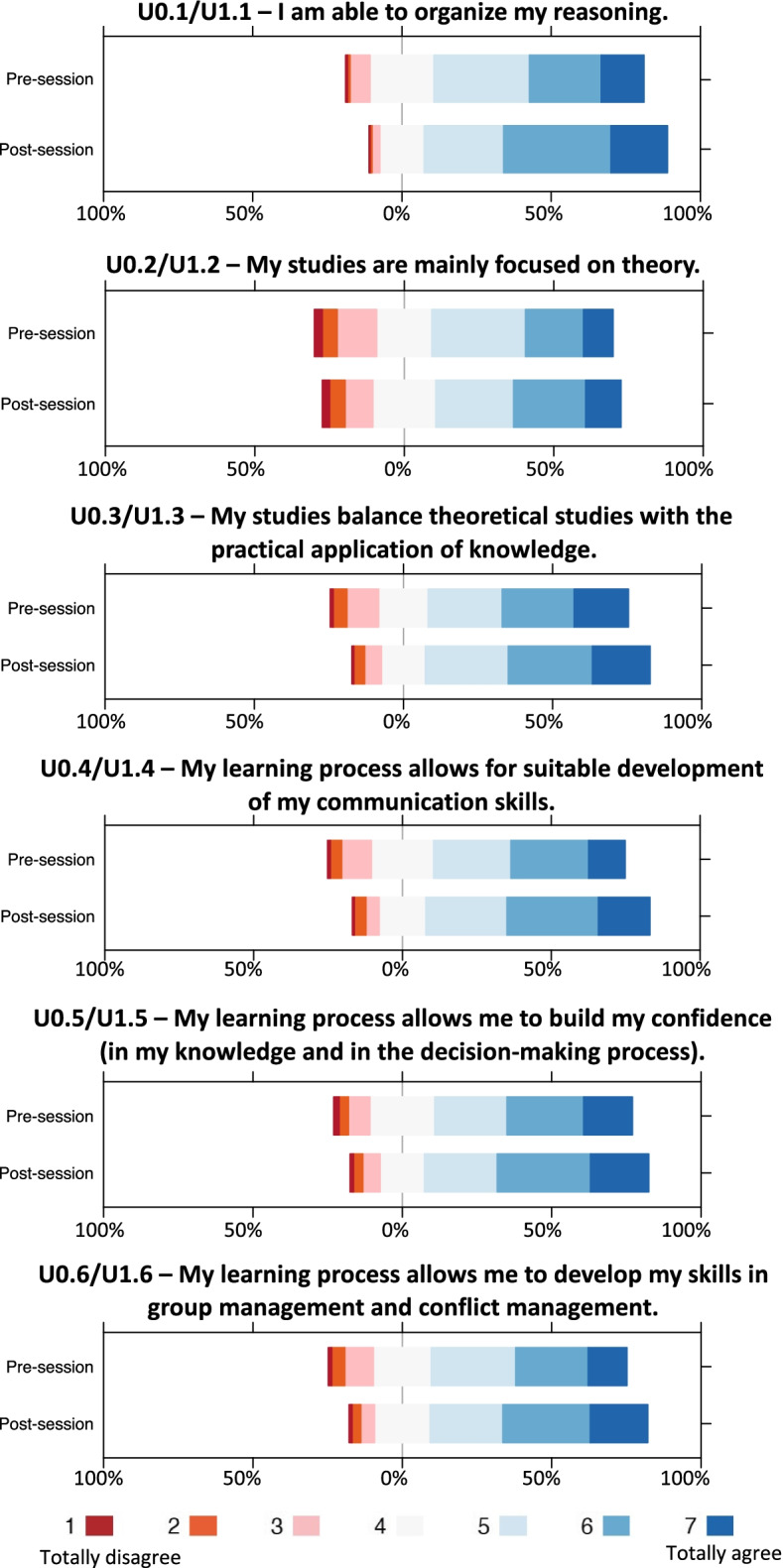
Perceptions of individual learning process on the pre- and post-session questionnaires. The x-axis displays the percentage of participants for each Likert response category. “Negative” responses (1 to 3 on a 7-point scale) are shown to the left of midline while “positive” responses (5 to 7 on a 7-point scale) are shown to the right. “Neutral” responses (4 on a 7-point scale) are shown straddling 0

On the post-session questionnaire, participants were asked to re-assess their individual learning process (Table 2). Paired items on the pre- and post-session questionnaires were compared to determine the impact of the VPS training experience (Table 3). With respect to individual learning process, 6/6 items showed significant increases on the post-session questionnaire: these included the balance between theoretical studies and the practical application of knowledge (*P* < 0.001), the development of communication skills (*P* < 0.001), the degree of confidence in the knowledge and decision-making process (*P* < 0.001) and the development of skills in group management and conflict management (*P* < 0.018) (Fig. 2).

**Table 2:** Results of the Post-Session Questionnaire (U1)

U1 | Post-Session with Body Interact simulator		Total (*n* = 617)	Medicine (*n* = 293)	Nursing (*n* = 324)	*t*-test
**Item scored from 1 = totally disagree to 7 = totally agree**		**Mean (SD)**			***p***
U1.1	I am able to organize my reasoning	5.50(1.11)	5.46(1.20)	5.54(1.01)	.39
U1.2	My studies are mainly focused on theory	4.83(1.46)	4.98(1.49)	4.69(1.42)	0.02
U1.3	My studies balance theoretical studies with the practical application of knowledge	5.28(1.34)	4.84(1.52)	5.67(0.99)	.001
U1.4	My learning process allows for suitable development of my communication skills	5.26(1.31)	5.03(1.49)	5.48(1.08)	.001
U1.5	My learning process allows me to build my confidence (in my knowledge and in the decision-making process)	5.30(1.36)	5.07(1.54)	5.51(1.14)	.001
U1.6	My learning process allows me to develop my skills in group management and conflict management	5.27(1.34)	5.14(1.51)	5.39(1.14)	.018
U1.7	In my course the contents are well integrated and connected with each other	5.40(1.25)	5.23(1.37)	5.57(1.10)	.001
U1.8	In my course there are opportunities to apply new learning to practical clinical cases	5.56(1.26)	5.29(1.39)	5.80(1.07)	.001
U1.9	In my course we have the opportunity to participate in clinical simulations	5.68(1.38)	5.36(1.58)	5.97(1.09)	.001
U1.10	In my course there is adequate training in communication techniques	5.22(1.45)	4.89(1.67)	5.53(1.14)	.001
U1.11	In my course there is discussion/debate of clinical decisions in a controlled learning environment	5.52(1.34)	5.20(1.51)	5.81(1.07)	.001
U1.12	My course helps me build the personal confidence necessary to function as a future professional	5.42(1.39)	5.18(1.59)	5.65(1.12)	.001
U1.13	I consider the teaching methods in my course appropriate	5.46(1.32)	5.19(1.49)	5.70(1.09)	.001
**Items scored from 1 = low satisfaction to 7 = high satisfaction**					
U1.14	Satisfaction level regarding the use of Body Interact as a new learning tool	6.27(1.01)	6.37(0.89)	6.17(1.10)	.015
U1.15	Satisfaction level regarding the use of a technological resource for learning	6.28(0.99)	6.38(0.97)	6.20(1.00)	.02
U1.16	Satisfaction level regarding the use of the Body Interact simulator	6.33(0.94)	6.46(0.89)	6.21(0.97)	.001
U1.17	Body Interact allowed me to bridge the learning gaps in the teaching process	6.06(1.05)	6.22(0.94)	5.92(1.11)	0.001
U1.18	Body Interact helped me to bridge the learning gaps in my own learning	6.08(1.06)	6.25(0.95)	5.92(1.14)	0.001
U1.19	Body Interact provided real feedback on my learning	6.12(1.07)	6.26(0.98)	5.98(1.13)	0.001
U1.20	Body Interact enabled me to identify individual weaknesses in my competencies	6.16(1.06)	6.32(0.93)	6.01(1.14)	0.001
U1.21	Body Interact gave me clinical experience (through simulation)	6.12(1.08)	6.30(0.99)	5.96(1.14)	0.001
U1.22	Body Interact validates the competencies I have already acquired	6.03(1.07)	6.19(1.04)	5.89(1.08)	0.001
U1.23	Body Interact helped me practice decision-making strategies	6.19(0.99)	6.33(0.85)	6.06(1.08)	0.001
U1.24	Body Interact turned clinical decision-making errors into a constructive learning process	6.25(0.98)	6.34(0.94)	6.18(1.00)	0.042
U1.25	Body Interact is an important learning tool	6.30(1.04)	6.50(0.91)	6.12(1.11)	0.001
**Items scored from 1 = not important in VPS training to 7 = highly important in VPS training**					
U1.26	Development of decision-making skills	6.26(0.93)	6.37(0.88)	6.16(0.95)	.006
U1.27	Development of independent-learning skills	6.22(0.91)	6.37(0.84)	6.09(0.96)	.001
U1.28	Simulation training	6.32(1.00)	6.47(0.89)	6.18(1.07)	.001
U1.29	Organization of reasoning and critical thinking	6.30(0.92)	6.44(0.84)	6.19(0.97)	.001
U1.30	Constructive feedback	6.27(0.97)	6.46(0.84)	6,10(1,06)	.001
U1.31	Ability to repeat clinical cases	6.31(0.95)	6.35(0.95)	6,26(0,94)	.230
U1.32	Complexity level of health conditions and clinical cases	6.30(0.91)	6.40(0.84)	6,22(0,96)	.013

**Table 3:** Responses to paired items on the pre-session and post-session questionnaires

**Empirical dimensions of VPS Instrument**	**Paired items **		**Mean difference (Mean U0, Mean U1)**	***t-test paired samples (****P****)***
(1) Individual Learning Process	U0.1	U1.1	0.37 (5.13, 5.50)	.001
	U0.2	U1.2	0.14 (4.69, 4.83)	.007
	U0.3	U1.3	0.23 (5.05, 5.28)	.001
	U0.4	U1.4	0.31 (4.95, 5.26)	.001
	U0.5	U1.5	0.24 (5.06, 5.30)	.001
	U0.6	U1.6	0.33 (4.94, 5.27)	.001
(2) Curricular Integration	U0.9	U1.7	0.37 (5.03, 5.40)	.001
	U0.10	U1.8	0.38 (5.18, 5.56)	.001
	U0.11	U1.9	0.43 (5.25, 5.68)	.001
	U0.12	U1.10	0.42 (4.80, 5.22)	.001
	U0.13	U1.11	0.46 (5.06, 5.52)	.001
	U0.14	U1.12	0.38 (5.04, 5.42)	.001
	U0.15	U1.13	0.42 (5.04, 5.46)	.001
(3a) VPS environment	U0.16	U1.14	0.39 (5.88, 6.27)	.001
	U0.17	U1.15	0.38 (5.90, 6.28)	.001
	U0.18	U1.16	0.39 (5.94, 6.33)	.001
(3b) VPS impact on student learning	U0.19	U1.17	0.24 (5.82, 6.06)	.001
	U0.20	U1.18	0.29 (5.79, 6.08)	.001
	U0.21	U1.19	0.31 (5.81, 6.12)	.001
	U0.22	U1.20	0.27 (5.89, 6.16)	.001
	U0.23	U1.21	0.30 (5.82, 6.12)	.001
	U0.24	U1.22	0.22 (5.81, 6.03)	.001
	U0.25	U1.23	0.29 (5.90, 6.19)	.001
	U0.26	U1.24	0.28 (5.97, 6.25)	.001
	U0.27	U1.25	0.34 (5.96, 6.30)	.001

### Impact of small-group VPS training on perceptions of curricular integration

**Fig. 3:**
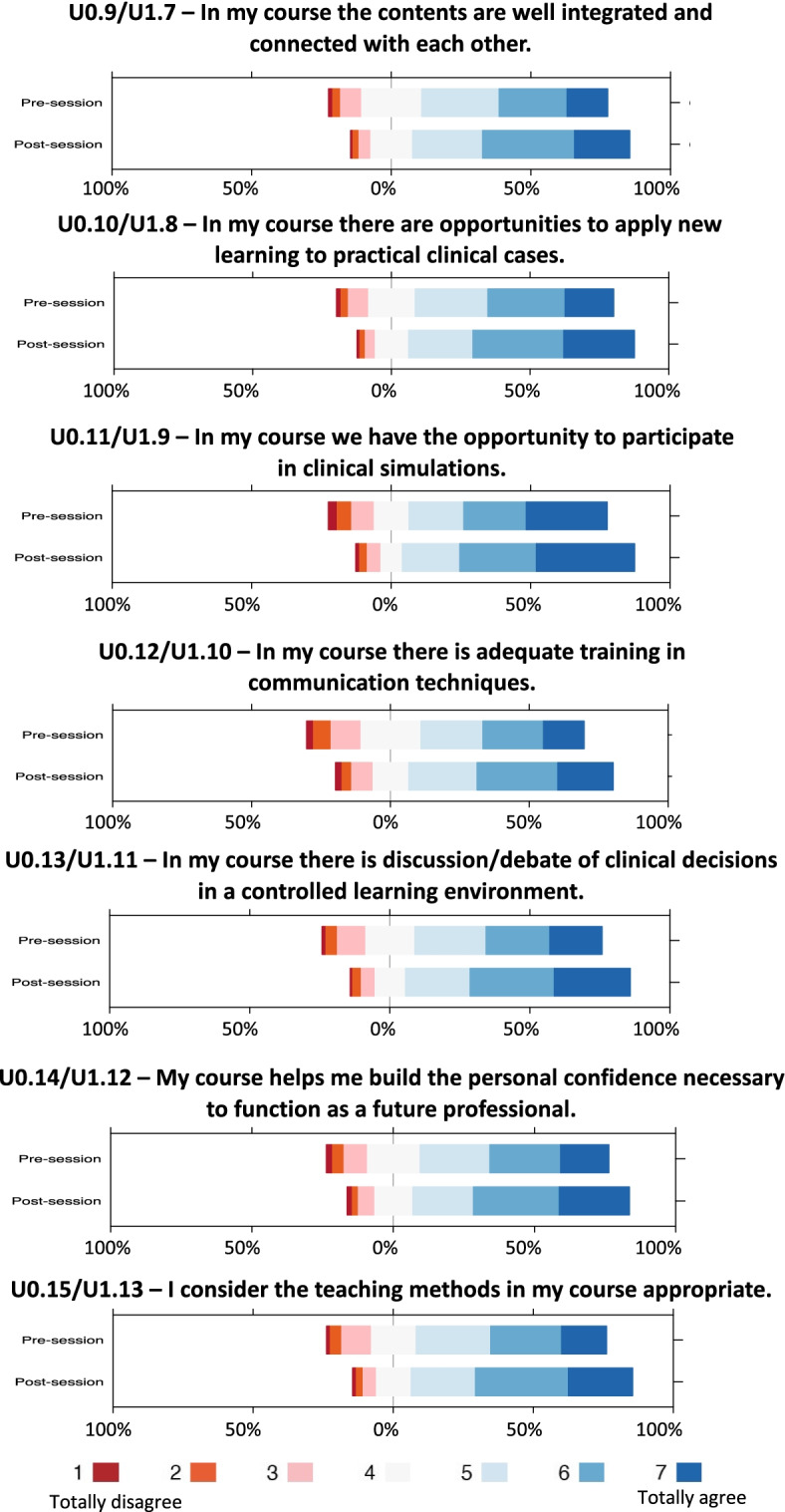
Perceptions of curricular integration on the pre- and post-session questionnaires. The x-axis displays the percentage of participants for each Likert response category. “Negative” responses (1 to 3 on a 7-point scale) are shown to the left of midline while “positive” responses (5 to 7 on a 7-point scale) are shown to the right. “Neutral” responses (4 on a 7-point scale) are shown straddling 0

On the post-session questionnaire, participants were asked to re-assess the degree of curricular integration in their courses (Table 2). All of the items relating to curricular integration (7/7) showed significant increases on the post-session questionnaire (Table 3); these included the integration of course contents (*P* < 0.001), opportunities to apply new learning to practical clinical cases (*P* < 0.001), opportunities to participate in clinical simulations (*P* < 0.001), training in communication techniques (*P* < 0.001), discussion of clinical decisions in a controlled learning environment (*P* < 0.001), and the building of confidence as a future professional (*P* < 0.001) (Fig. 3).

### Assessment of the small-group VPS training experience

On the post-session questionnaire (Table 2), participants reported a high level of satisfaction with the VPS as a learning tool (*M* = 6.27, *SD* = 1.01) and with the use of a technological resource for learning (*M* = 6.28, *SD* = 0.99). They reported that the VPS helped fill gaps in the teaching process (*M* = 6.06, *SD* = 1.05) and in their own individual learning (*M* = 6.08, *SD* = 1.06). They also felt that it provided real feedback on learning (*M* = 6.12, *SD* = 1.07) and identified individual weaknesses in their competencies (*M* = 6.16, *SD* = 1.06). Finally, students felt that the VPS provided clinical experience through simulation (*M* = 6.12, *SD* = 1.08), helped practice decision-making strategies (*M* = 6.19, *SD* = 0.99), and turned clinical decision-making errors into a constructive learning process (*M* = 6.25, *SD* = 0.98). Overall, participants considered the VPS to be an important learning tool (*M* = 6.30, *SD* = 1.04). When responses from nursing and medical students were compared, medical students reported greater satisfaction with the use of the VPS (*P* < 0.001) and with its ability to bridge learning gaps (*P* < 0.001), provide feedback on learning (*P* < 0.001) and identify weaknesses (*P* < 0.001)(Table 2).

### The impact of small-group VPS training on nursing versus medical students

Responses from nursing and medical students were analysed separately to determine whether the impact of the small-group VPS training experience differed between the two groups (Supplementary Table 3). No significant differences were noted for items relating to individual learning process. One item relating to curricular integration showed a significant difference (U0.11-U1.9: “In my course we have the opportunity to participate in clinical simulations”), with medical students reporting greater positive change relative to nursing students (Supplementary Table 3). No other items relating to curricular integration showed significant differences between the two courses.

### Internal behaviour of the instrument

Exploratory factor analysis was used to examine the internal behaviour of the survey instrument before and after small-group VPS training. On the pre-session questionnaire (U0), three factors were defined (Supplementary Tables 4 and 5): factor C1 represented “curricular integration” and was most impactful while factor C3 represented “focus on theory” and was most specific. An additional factor, C2, was loaded with only one item (U0.12 – “In my course there is adequate training in communication techniques”), which was split over C1 and C2. On the post-session questionnaire, the matched item (U1.10) loaded primarily onto factor C1. This 3-factor model accounted for 60.2% of the variance on the pre-session questionnaire and 73.8% of the variance on the post-session questionnaire.

## Discussion

In this multi-centre study, nursing and medical students from 11 institutions participated in small-group VPS training sessions supervised by clinical tutors. Perceptions of individual learning process and curricular integration were assessed before and after the VPS training experience. Results demonstrate that small-group VPS training had a positive impact on individual learning process, including the balance between theoretical studies and the practical application of knowledge, the development of communication skills, and the development of group and conflict management skills. Small-group VPS training also improved perceived curricular integration, including the degree of integration of course contents, opportunities to apply learning to clinical cases and opportunities to participate in clinical simulations.

At baseline, nursing and medical students differed significantly in terms of individual learning process and curricular integration. Nursing students reported less focus on theory and more clinical and simulation experience. They also reported more opportunities to train non-technical skills such as communication and group and conflict management. Despite these underlying differences, the impact of the intervention was similar across the two groups, suggesting that VPS training can be beneficial in well-integrated courses as well as in more theoretical courses.

Medical students had higher expectations of the VPS training experience and rated the experience more highly afterwards. Studies suggest that pre-clinical medical students and pre-clinical nursing students have different perceived learning needs: medical students are focused on learning to gather clinical information and formulating a differential diagnosis [12] while nursing students are focused on acquiring knowledge and technical skills [13]. VPS training aligns closely with the learning needs of medical students, and this may explain their higher expectations and evaluations.

It is important to note that participants in this study were exposed to small-group VPS training, and not large-group or individual VPS training. Studies have shown that the small-group environment contributes to active learning, enhances deep learning, and helps learners identify weaknesses [14]. It also encourages the development of soft skills, such as communication, teamwork, and group and conflict management [14]. Thus, the small-group environment may have contributed to the benefits of the VPS training experience. A recent study from Japan reported that large-group VPS training improved clinical reasoning scores [15]. The authors did not assess participant perceptions of the experience nor its impact on curricular integration. Studies comparing VPS training in small-groups, large-groups and individually are lacking, however it is likely that the impact of VPS training will differ between these modalities.

Most of the participants in the present study were in their “pre-clinical” years (years 1–2 for nursing students and 1–3 for medical students), where the focus is on theoretical learn and patient contact is more limited. Additional studies will be required to determine whether VPS training has a similar impact on students in their “clinical” years, when there is usually more exposure to the clinical environment.

In conclusion, the results of this study demonstrate that small-group VPS training contributed positively to perceptions of individual learning process and curricular integration amongst nursing and medical students. It also improved opportunities to train key non-technical skills, such as communication and group and conflict management. Further studies will be required to determine whether these benefits translate into improvements in clinical reasoning, decision-making and non-technical skill acquisition.

### Limitations

The study has several important limitations. Firstly, variation existed between study sites in terms of (1) the clinical cases used in the VPS sessions, (2) whether the VPS session was integrated into the curriculum or offered as a stand-alone session and (3) the language of instruction. These variations were expected given the pragmatic design of the study. In spite of these differences, similar results were observed across institutions, suggesting a limited effect of these variables on study results. Secondly, VPS training occured in small groups supervised by a clinical tutor, so results cannot be extrapolated to unsupervised VPS training or to individual or large-group VPS training. Finally, the study did not assess hard outcomes such as clinical reasoning, decision-making or non-technical skills.

## Conclusions

Small-group VPS training offers an accessible simulation experience that is well-received by students and is perceived to improve individual learning process and curricular integration. These results indicate that small-group VPS training is a valuable educational tool that imparts some of the benefits of high-fidelity simulation and clinical training. Future studies should compare VPS training in small-groups, large groups and individually as well as quantifying the impact of VPS training on clinical reasoning, decision-making and non-technical skill acquisition.

## Supplementary Information

**Additional file 1. **Supplementary methods.

**Additional file 2: Supplementary Figure 1.** Year of study of participating medical and nursing students. **Supplementary Figure 2.** Perceptions of individual learning process amongst nursing and medical students on the pre-session questionnaire. The x-axis displays the percentage of participants for each Likert response category. “Negative” responses (1 to 3 on a 7-point scale) are shown to the left of midline while “positive” responses (5 to 7 on a 7-point scale) are shown to the right. “Neutral” responses (4 on a 7-point scale) are shown straddling 0. **Supplementary Figure 3.** Perceptions of curricular integration amongst nursing and medical students on the pre-session questionnaire. The x-axis displays the percentage of participants for each Likert response category. “Negative” responses (1 to 3 on a 7-point scale) are shown to the left of midline while “positive” responses (5 to 7 on a 7-point scale) are shown to the right. “Neutral” responses (4 on a 7-point scale) are shown straddling 0.

**Additional file 3: Supplementary Table 1.** Pre-session questionnaire (U0). **Supplementary Table 2.** Post-session questionnaire (U1). **Supplementary Table 3**. Comparison of pre-session and post-session responses of, subdivided by course of study (nursing versus medicine). **Supplementary Table 4.** Factor analysis for pre-session questionnaire (U0). **Supplementary Table 5.** Factor analysis for post-session questionnaire (U1).

## Data Availability

The datasets analysed during the current study are available from the corresponding author on reasonable request.

## Abbreviations

VP: Virtual Patient
VPS: Virtual Patient Simulator
HFS: High Fidelity Simulator
