# Supplementary material for: The impact of small-group virtual patient simulator training on perceptions of individual learning process and curricular integration: a multicentre cohort study of nursing and medical students
Source: BMC Med Educ. 2022 May 16;22:375. doi: 10.1186/s12909-022-03426-3 (PMC9109952; doi:10.1186/s12909-022-03426-3)
Supplement: Supplementary file 1 — Additional file 1. Supplementary methods. [file 12909_2022_3426_MOESM1_ESM.pdf]

STUDY PROTOCOL | STUDENT
June 2019 - July 2020

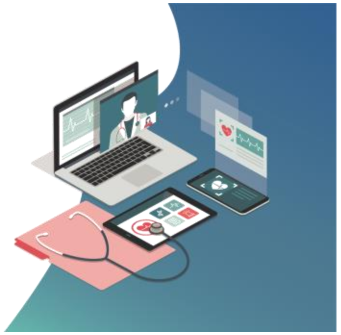

Study protocol goal is to <u>standardize implementation</u> accross participants, providing a step-by-step of implementation phase, in order to inform every participant university/teacher involved of how the study must be implemented

Observations:
- Total of clinical case in this protocol is only three cases (1 experimental + 2 for development in classes).
- If school or tutor wants to continue the use of VPS after the performance of the 3 cases, protocol is concluded. No more than 3 cases must be performed.
- Suggested number minimum and maximum of students: 5 - 10 students.
- Students and tutor e-mail in pre and post questionnaire must be the same, otherwise it won't be possible to make a match.
- **<u>In order for data of all universities be considered valid, every participant must fullfill the questionnaire in due time.</u>**

	Title	Details		Goal | Explanation
		Target	Expected timed	
1	Brief introduction of the platform*	_	10 min	Tutor briefly presents simulator, in a non judgemental, allowing for students to creat their own impressions. We suggest this to be done orally and with this video https://vimeo.com/204523314/8753df3383
2	Pre-session VPS questionnaire - **Student version***	Student	5 min	Fullfilment of online inquiry that is sent to students e-mail on that day, by VPS. This should be done before teacher gets hands-on the simulator. We suggested that students fullfill the questionnaire immediately on their mobile phone.
	**Important reminder: Check if inquiry is on spam box**	_	_	_
3	Experimental case resolution		Tutor criteria	Tutor presents simulator with the goal of showing how the tool works and where different things can be found. We suggested that this would be done by interacting with students, asking questions the case resolution.<br>Tutor is free to do the experimental case that he wants (if a suggestion is required, our would be #1). The approach and development of the clinical case will be done how the teacher intends.
3	Repetition of experimental case resolution		Optional	The intention for this repetition is to provide students with skills to manage case resolution, assimilation of keys features of simulator and to be at ease with the simulator.
4	Development of <u>two VPS clinical cases</u>*		Tutor criteria	There are no specific guidelines to case development, presentation and pace of resolution. Schools/Tutors are free to guide any two Body Interact clinical cases as pleased/ as they usually do.
5	Post-session VPS inquiry*<br>**Important reminder: Check if inquiry is on spam box**	Student	5 min	Fullfilment of online inquiry that is sent to students e-mail on that day, by VPS.
