# Supplementary material for: The impact of small-group virtual patient simulator training on perceptions of individual learning process and curricular integration: a multicentre cohort study of nursing and medical students
Source: BMC Med Educ. 2022 May 16;22:375. doi: 10.1186/s12909-022-03426-3 (PMC9109952; doi:10.1186/s12909-022-03426-3)

Supplementary Figures

Mestre et al., "*Impact of Small-Group Virtual Patient Simulator Training on individual learning process and curricular integration: a multicentre cohort study of nursing and medical students*."

Supplementary Figure 1

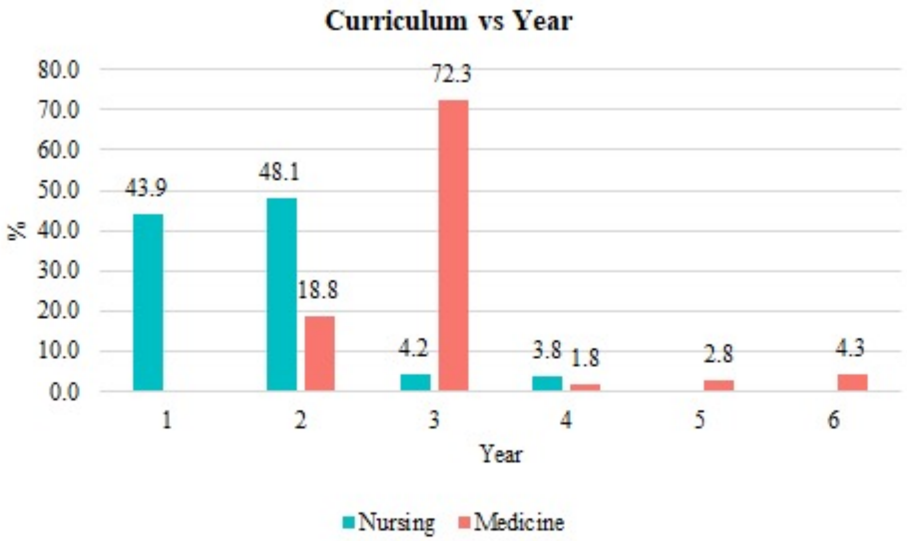

Supplementary Figure 2

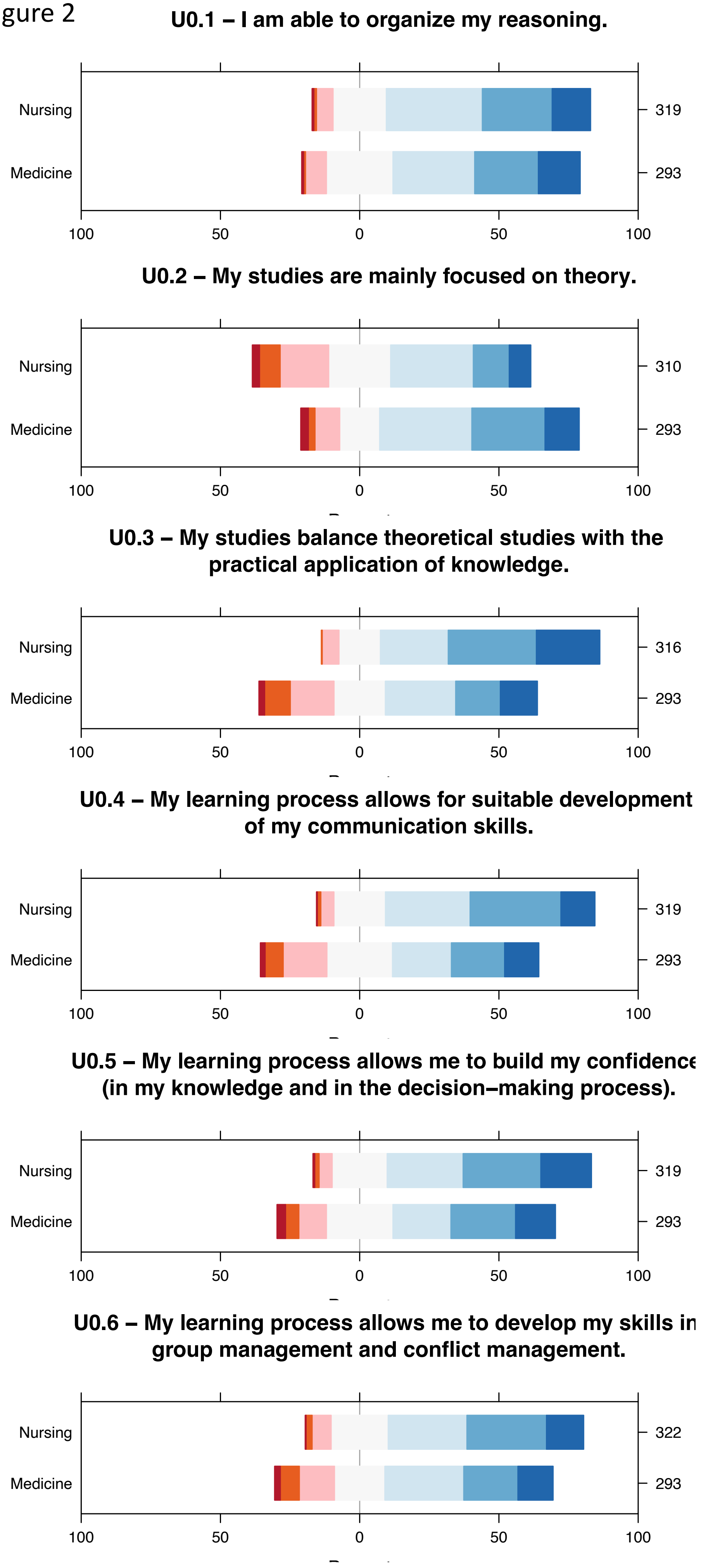

Supplementary Figure 3

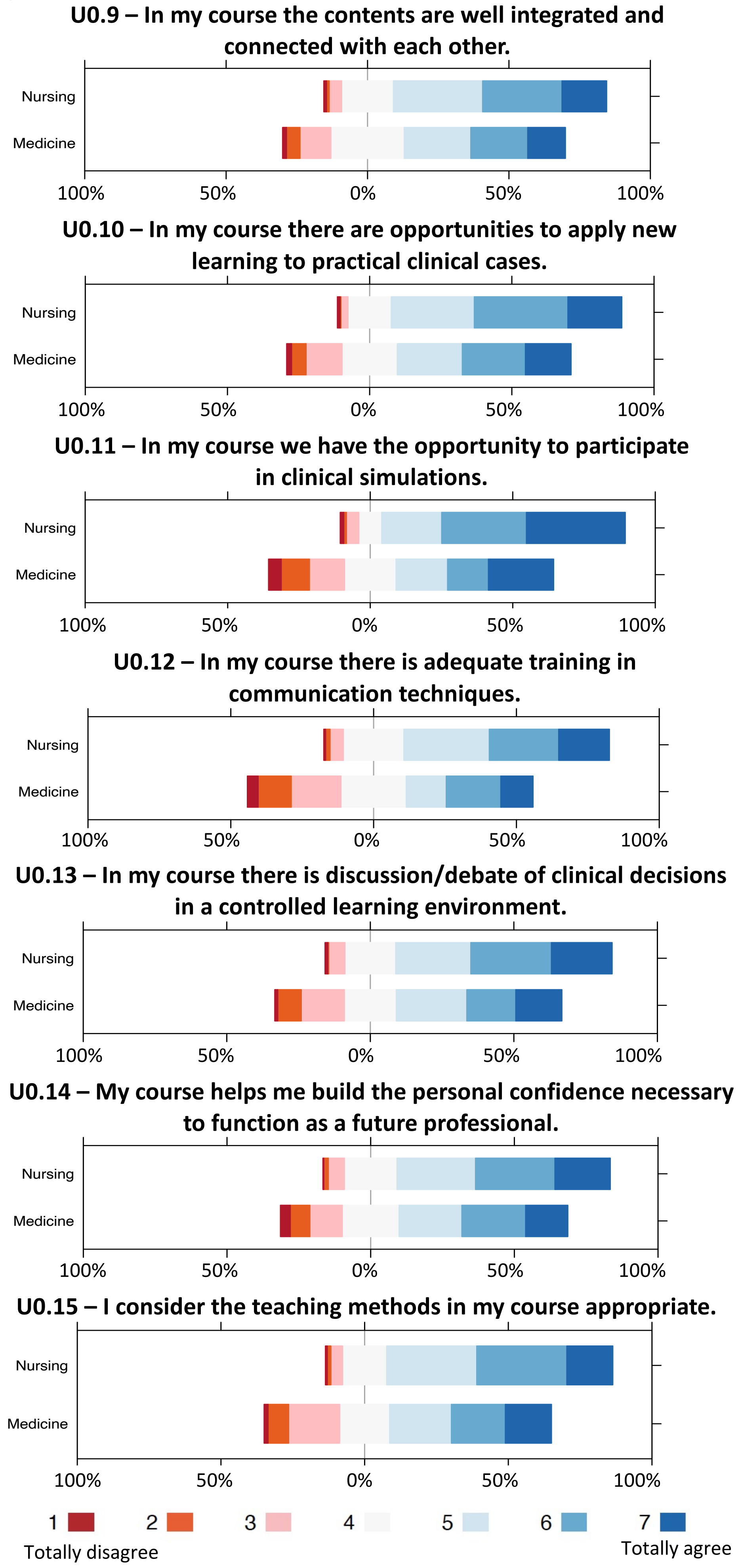

Supplement: Supplementary file 2 — Additional file 2: Supplementary Figure 1. Year of study of participating medical and nursing students. Supplementary Figure 2. Perceptions of individual learning process amongst nursing and medical students on the pre-session questionnaire. The x-axis displays the percentage of participants for each Likert response category. “Negative” responses (1 to 3 on a 7-point scale) are shown to the left of midline while “positive” responses (5 to 7 on a 7-point scale) are shown to the right. “Neutral” responses (4 on a 7-point scale) are shown straddling 0. Supplementary Figure 3. Perceptions of curricular integration amongst nursing and medical students on the pre-session questionnaire. The x-axis displays the percentage of participants for each Likert response category. “Negative” responses (1 to 3 on a 7-point scale) are shown to the left of midline while “positive” responses (5 to 7 on a 7-point scale) are shown to the right. “Neutral” responses (4 on a 7-point scale) are shown straddling 0. [file 12909_2022_3426_MOESM2_ESM.pdf]
