# Supplementary material for: The impact of small-group virtual patient simulator training on perceptions of individual learning process and curricular integration: a multicentre cohort study of nursing and medical students
Source: BMC Med Educ. 2022 May 16;22:375. doi: 10.1186/s12909-022-03426-3 (PMC9109952; doi:10.1186/s12909-022-03426-3)
Supplement: Supplementary file 3 — Additional file 3: Supplementary Table 1. Pre-session questionnaire (U0). Supplementary Table 2. Post-session questionnaire (U1). Supplementary Table 3. Comparison of pre-session and post-session responses of, subdivided by course of study (nursing versus medicine). Supplementary Table 4. Factor analysis for pre-session questionnaire (U0). Supplementary Table 5. Factor analysis for post-session questionnaire (U1). [file 12909_2022_3426_MOESM3_ESM.pdf]

Supplementary tables

Mestre et al., "*Impact of Small-Group Virtual Patient Simulator Training on individual learning process and curricular integration: a multicentre cohort study of nursing and medical students.*"

Supplementary table 1: Pre-session questionnaire

U0 | Pre-Session with Body Interact simulator	
**On a scale from 1-7 (1 = totally disagree and 7 = totally agree), rate each of the following items regarding your current individual learning process:**	
U0.1	I am able to organize my reasoning.
U0.2	My studies are mainly focused on theory.
U0.3	My studies balance theoretical studies with the practical application of knowledge.
U0.4	My learning process allows for suitable development of my communication skills.
U0.5	My learning process allows me to build my confidence (in my knowledge and in the decision-making process).
U0.6	My learning process allows me to develop my skills in group management and conflict management.
U0.7	My clinical experience is appropriate for my knowledge level.
U0.8	My simulation experience is appropriate for my knowledge level.
**On a scale from 1-7 (1 = totally disagree and 7 = totally agree), rate each of the following items regarding your current pedagogical process:**	
U0.9	In my course the contents are well integrated and connected with each other.
0.10	In my course there are opportunities to apply new learning to practical clinical cases.
U0.11	In my course we have the opportunity to participate in clinical simulations.
U0.12	In my course there is adequate training in communication techniques.
U0.13	In my course there is discussion/debate of clinical decisions in a controlled learning environment.
U0.14	My course helps me build the personal confidence necessary to function as a future professional.
U0.15	I consider the teaching methods in my course appropriate.
**On a scale from 1 – 7 (1 = low expectation and 7 = high expectation), rate each of the following items regarding your expectations about the use of Body Interact simulator:**	
U0.16	Expectations regarding the use of a new learning tool.
U0.17	Expectations regarding the use of a technological resource for learning.
U0.18	Expectations regarding using Body Interact as a simulator.
**On a scale from 1 – 7 (1 = totally disagree and 7 = totally agree), rate each of the following items regarding your learning experience expectations with the Body Interact simulator:**	
U0.19	I expect that Body Interact will help to fill in the learning gaps in the teaching process.
U0.20	I expect that Body Interact will help to fill in the individual gaps in my current learning.
U0.21	I expect that Body Interact will provide real feedback on my learning.
U0.22	I expect that Body Interact will help me identify individual weaknesses in my competencies.
U0.23	I expect that Body Interact will give me clinical experience (through simulation).
U0.24	I expect that Body Interact will validate the competencies I have already acquired (through simulation).
U0.25	I expect that Body Interact will help me practice decision-making strategies.
U0.26	I expect that Body Interact transform clinical decision-making errors into a constructive learning process.
U0.27	I expect that that Body Interact will become an important learning tool.

Supplementary table 2: Post-session questionnaire

**U1 | Post-Session with Body Interact simulator**	
**On a scale from 1-7 (1 = totally disagree and 7 = totally agree), rate each of the following items regarding your current individual learning process after your experience with the Body Interact simulator:**	
U1.1	I am able to organize my reasoning.
U1.2	My studies are mainly focused on theory.
U1.3	My studies balance theoretical studies with the practical application of knowledge.
U1.4	My learning process allows for suitable development of my communication skills.
U1.5	My learning process allows me to build my confidence (in my knowledge and in the decision-making process).
U1.6	My learning process allows me to develop my skills in group management and conflict management.
**On a scale from 1 a 7 (1 = totally disagree and 7 = totally agree), rate each of the following items regarding any gaps in your current pedagogical process after your experience with the Body Interact simulator:**	
U1.7	In my course the contents are well integrated and connected with each other.
U1.8	In my course there are opportunities to apply new learning to practical clinical cases.
U1.9	In my course we have the opportunity to participate in clinical simulations.
U1.10	In my course there is adequate training in communication techniques.
U1.11	In my course there is discussion/debate of clinical decisions in a controlled learning environment.
U1.12	My course helps me build the personal confidence necessary to function as a future professional.
U1.13	I consider the teaching methods in my course appropriate.
**On a scale from 1 – 7 (1 = low satisfaction and 7 = high satisfaction), rate each of the following items regarding your satisfaction with the use of the Body Interact simulator:**	
U1.14	Satisfaction level regarding the use of Body Interact as a new learning tool.
U1.15	Satisfaction level regarding the use of a technological resource for learning.
U1.16	Satisfaction level regarding the use of the Body Interact simulator.
**On a scale from 1 – 7 (1 = totally disagree and 7 = totally agree), rate each of the following items regarding your learning experience with the Body Interact simulator:**	
U1.17	Body Interact allowed me to bridge the learning gaps in the teaching process.
U1.18	Body Interact helped me to bridge the learning gaps in my own learning.
U1.19	Body Interact provided real feedback on my learning.
U1.20	Body Interact enabled me to identify individual weaknesses in my competencies.
U1.21	Body Interact gave me clinical experience (through simulation).
U1.22	Body Interact validates the competencies I have already acquired.
U1.23	Body Interact helped me practice decision-making strategies.
U1.24	Body Interact turned clinical decision-making errors into a constructive learning process.
U1.25	Body Interact is an important learning tool.
**On a scale from 1 -7 (1 = not important and 7 = highly important), rate each of the following items regarding the level of importance you attach to training that uses simulation with virtual patients:**	
U1.26	Development of decision-making skills.
U1.27	Development of independent-learning skills.
U1.28	Simulation training.

U1.29	Organization of reasoning and critical thinking.
U1.30	Constructive feedback.
U1.31	Ability to repeat clinical cases.
U1.32	Complexity level of health conditions and clinical cases.

Supplementary Table 3

Empirical dimensions of VPS Instrument	Paired items of VPS Instrument		Nursing students	Medical students	*t-test paired samples**
(1) Individual Learning Process	U0.1	U1.1	0.38 (0.95)	0.37 (1.23)	0.949
	U0.2	U1.2	0.32 (1.23)	-0.03 (1.46)	0.035
	U0.3	U1.3	0.18 (1.03)	0.27 (1.40)	0.715
	U0.4	U1.4	0.23 (1.04)	0.41 (1.26)	0.429
	U0.5	U1.5	0.21 (1.18)	0.25 (1.37)	0.254
	U0.6	U1.6	0.26 (1.09)	0.41 (1.43)	0.450
(2) Curricular Integration	U0.9	U1.7	0.31 (1.11)	0.43 (1.45)	0.275
	U0.10	U1.8	0.34 (1.15)	0.41 (1.43)	0.626
	**U0.11**	**U1.9**	**0.23 (1.19)**	**0.66 (1.55)**	**0.001**
	U0.12	U1.10	0.33 (1.14)	0.55 (1.45)	0.374
	U0.13	U1.11	0.44 (1.20)	0.48 (1.49)	0.052
	U0.14	U1.12	0.34 (1.09)	0.43 (1.45)	0.692
	U0.15	U1.13	0.36 (1.13)	0.48 (1.46)	0.142

Boldfaced values - Variables with P < 0.01

Supplementary Table 4 – Factor analysis for pre-session questionnaire (U0).

Item/ Component	U0 | Pre-session questionnaire		
	**C1**	**C2**	**C3**
**U0.1**	0,482	0,305	0,523
**U0.2**	0,120	-0,173	0,722
**U0.3**	0,576	0,506	-0,184
**U0.4**	0,611	0,528	0,179
**U0.5**	0,615	0,521	0,257
**U0.6**	0,633	0,452	0,253
**U0.7**	0,562	0,468	0,034
**U0.8**	0,593	0,363	-0,041
**U0.9**	0,630	0,436	-0,019
**U0.10**	0,672	0,420	-0,224
**U0.11**	0,558	0,472	-0,339
**U0.12**	0,581	0,585	-0,098
**U0.13**	0,612	0,505	-0,144
**U0.14**	0,634	0,559	0,043
**U0.15**	0,613	0,554	-0,028
**U0.16**	0,744	-0,383	-0,047
**U0.17**	0,759	-0,381	-0,025
**U0.18**	0,713	-0,430	-0,037
**U0.19**	0,754	-0,450	0,026
**U0.20**	0,772	-0,446	-0,028
**U0.21**	0,747	-0,481	-0,010
**U0.22**	0,759	-0,427	-0,025
**U0.23**	0,726	-0,453	-0,010
**U0.24**	0,764	-0,422	0,014
**U0.25**	0,772	-0,463	-0,014
**U0.26**	0,767	-0,466	-0,027
**U0.27**	0,736	-0,510	-0,042
**Extraction Method**	Principal Component Analysis		
**Total variance explained C1-C3**	60,199%		

Supplementary Table 5 – Factor analysis for post-session questionnaire (U1).

Item/ Component	U1 | Post-session questionnaire		
	**C1**	**C2**	**C3**
**U1.1**	0,577	0,352	0,335
**U1.2**	0,162	0,026	0,882
**U1.3**	0,603	0,549	-0,033
**U1.4**	0,641	0,529	0,118
**U1.5**	0,693	0,527	0,088
**U1.6**	0,654	0,509	0,142
**U1.7**	0,668	0,481	0,004
**U1.8**	0,713	0,494	-0,126
**U1.9**	0,608	0,489	-0,246
**U1.10**	0,659	0,557	-0,031
**U1.11**	0,678	0,521	-0,143
**U1.12**	0,720	0,518	-0,029
**U1.13**	0,674	0,526	-0,126
**U1.14**	0,754	-0,448	-0,052
**U1.15**	0,759	-0,438	-0,062
**U1.16**	0,715	-0,459	-0,006
**U1.17**	0,761	-0,438	0,045
**U1.18**	0,781	-0,462	0,053
**U1.19**	0,721	-0,447	0,004
**U1.20**	0,716	-0,469	0,034
**U1.21**	0,733	-0,464	-0,015
**U1.22**	0,757	-0,402	0,012
**U1.23**	0,752	-0,415	-0,038
**U1.24**	0,751	-0,435	-0,038
**U1.25**	0,736	-0,491	-0,052
**Extraction Method**	Principal Component Analysis		
**Total variance explained C1-C3**	73,799%		
